# Hearing impairment improved after treatment with asfotase alfa in a case of perinatal hypophosphatasia

**DOI:** 10.1016/j.ymgmr.2020.100612

**Published:** 2020-06-06

**Authors:** Rie Chida-Naomiya, Masaru Shimura, Ryuhei Nagao, Atsushi Kumada, Hisashi Kawashima

**Affiliations:** Department of Pediatrics and Adolescent Medicine, Tokyo Medical University, 6-7-1 Nishishinjuku, Shinjuku-ku, Tokyo 160-0023, Japan

**Keywords:** Hypophosphatasia, Perinatal hypophosphatasia, Hearing impairment, Deafness, Asftase alfa

## Abstract

Hearing impairment is a neurological symptom of hypophosphatasia (HPP), which leads to a reduced quality of life. However, the pathomechanism of hearing impairment and the effects of asfotase alfa enzyme replacement therapy on hearing function in HPP have not been clarified. Here we report a case and present clinical data of a patient with perinatal HPP whose hearing impairment improved after asfotase alfa treatment.

## 1 Introduction

Hypophosphatasia (HPP, OMIM #241500) is an inborn error of metabolism characterized by defective bone mineralization due to deficient activity of the tissue-nonspecific isoenzyme of alkaline phosphatase (TNSALP). Asfotase alfa administration, enzyme replacement therapy, improves the symptoms of HPP, such as bone mineralization, growth, respiratory function, and mobility, and significantly improves survival rate [1]. As a result, the goal of HPP treatment is not only an improvement in survival rate but also in quality of life [2].

Hearing impairment, which can be conductive, sensorineural, or mixed, is a neurological symptom of HPP and develops in about 30% of HPP patients [3]. Hearing impairment decreases the quality of life and causes communication difficulties. Few studies to date have reported on the effects of asfotase alfa on hearing impairment, and its pathology in HPP is not well understood. We here report a case of a patient with perinatal HPP and hearing impairment treated with asfotase alfa.

## 2 Case report

A Japanese boy showed intrauterine growth restriction and shortened bones of the extremities on echocardiography during gestation. He was born at 38 weeks of gestation by planned cesarean section. His birth weight was 2465 g (−0.8 SD), height was 45.0 cm (−2.0 SD), chest circumference was 29.9 cm (−0.9 SD), and head circumference was 32.2 cm (−0.8 SD). He was admitted to the Neonatal Intensive Care Unit soon after birth with respiratory distress syndrome. X-ray displayed thin ribs with poor ossification, shortening of the long tubular bones, and poor cranial bone formation. He had a narrow chest, due to the skeletal malformation, which caused respiratory failure and tracheobronchomalacia (TBM). He was sedated and put on mechanical ventilation with a high peak inspiratory pressure of 20–25 cmH_2_O and positive end expiratory pressure of 5–8 cmH_2_O. Laboratory analyses demonstrated a low serum alkaline phosphatase (ALP) level at 12 IU/L (reference range: 530–1610 IU/L) and a slightly increased corrected serum calcium level of 11.8 mg/dL (reference range: 8.8–11.3 mg/dL). Urinary calcium/urinary creatinine ratio was 0.43 mg/mg (reference range: < 0.21 mg/mg). His parents were asymptomatic; however, both also had low serum ALP levels (mother: 113 IU/L; father: 95 IU/L (reference range: 104–338 U/L)). Owing to the clinical manifestations, examination results, and family history, the patient was clinically diagnosed as having perinatal lethal HPP. Subsequently, genetic analysis of the *ALPL* gene was performed after obtaining informed consent from the parents. Sanger sequencing identified compound heterozygous variants in the *ALPL* including the previously reported pathogenic variants c.1559delT (p.Leu520Argfs) and c.1276G > A (p.Gly426Ser) (NM_000478.5) [4,5], confirming the diagnosis. Asfotase alfa administration was started at the age of 5 years and his weight was 4600 g (−4.9 SD), height was 58.5 cm (−11.2 SD), and head circumference was 41.5 cm (−6.3 SD). Laboratory analyses demonstrated a markedly low ALP level at 9 IU/L and serum calcium level of 10.4 mg/dL. He showed hypomineralization of bones, TBM with mechanical ventilation, and renal calculus. After 5 months of treatment, bone mineralization was observed on radiography. His height, weight, and chest circumference gradually increased. TBM gradually improved and he was able to remove his respirator and go out of the hospital in his wheelchair at the age of 9 years and 8 months.

**Fig. 1:**
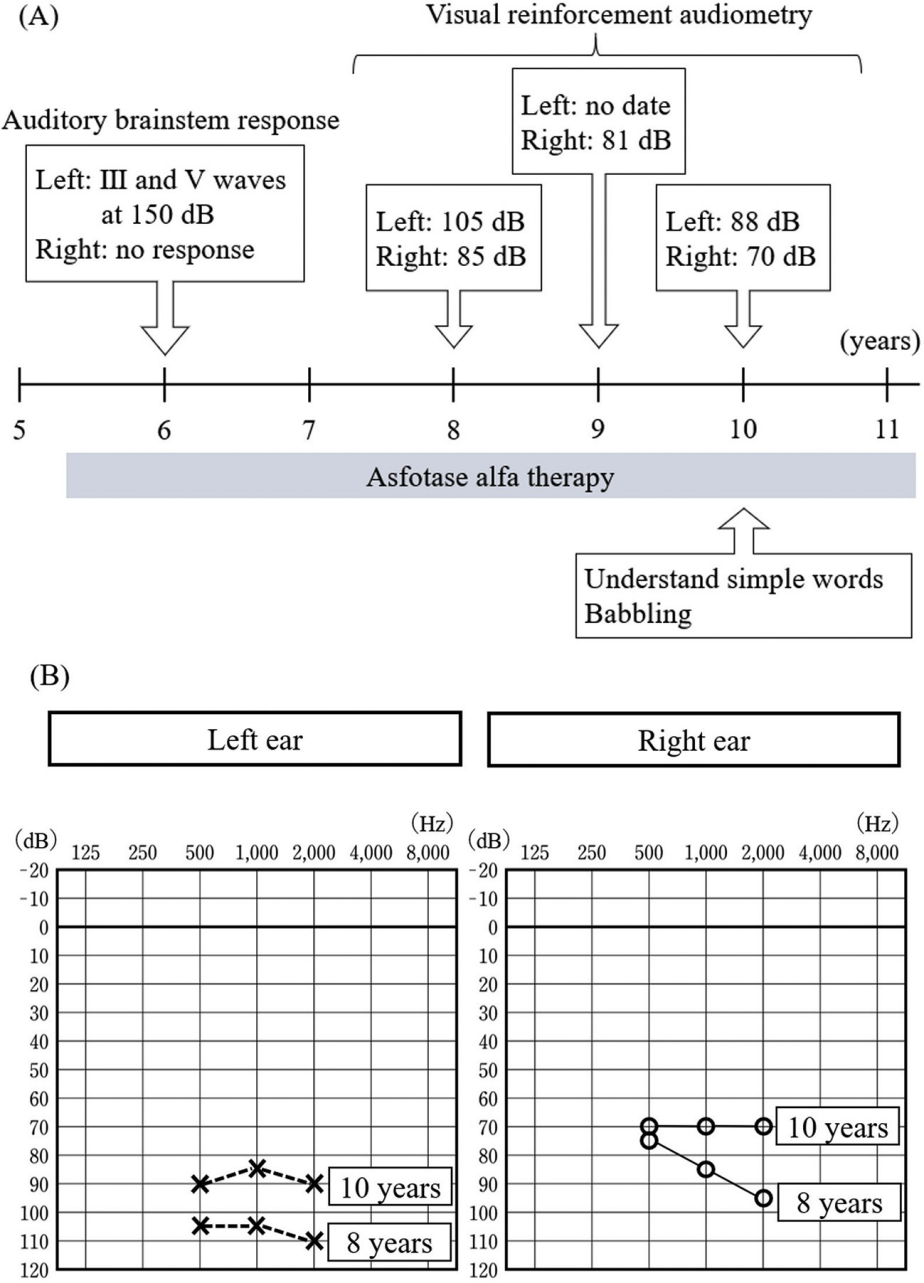
Auditory evaluation over the course of asfotase alfa therapy. Panel (A) shows the timeline of auditory evaluation over the course of asfotase alfa therapy and (B) shows hearing impariment improvement.

Audiological evaluation was carried out several times over the course of therapy. Hearing impairment improved after asofotase alfa administration. Initial evaluation of the auditory brainstem response at 6 years demonstrated that there was no response in the right ear, and that waves III and IV were detected at 150 dB in the left ear. The patient was diagnosed as having profound hearing impairment. He was re-evaluated by behavioral observation audiometry and visual reinforcement audiometry at the age of 8 years. On behavioral observation audiometry, he did not respond to 80 dB sounds, nor loud sounds of a drum. Visual reinforcement audiometry demonstrated improvement of his hearing capacity. Following these results, he started using hearing aids at 8 years of age. His hearing ability gradually improved, and he began to respond to sounds without hearing aids. More babbling was observed, and it appeared that he could understand simple words (i.e., bye-bye) together with gestures. The most recent evaluation with visual reinforcement audiometry at the age of 10 years demonstrated mild hearing impairment, which was a significant improvement of the hearing capacity from profound hearing impairment at the initial evaluation (Fig. 1).

## 3 Discussion

The pathology of hearing impairment in HPP is not well understood. One study has reported defects on bones that are thought to be associated with HPP. Okazaki et al. [6] reported a case of a patient with perinatal HPP with auditory ossicle hypoplasia as well as inner ear fusion and hypoplasia. In terms of middle ear development, auditory ossicles develop from the first and second arche mesenchymes and the 3 blastemal ossicles become cartilaginous by 8 weeks of gestation. Then, ossification of the ossicles begins at 16 weeks of gestation and is complete at about 24 weeks [7]. For HPP patients, ossification of the ossicles may be disrupted, leading to ossicle hypoplasia. Abnormalities of the auditory ossicles and the inner ear are known causes of deafness [8]. Hearing impairment in HPP might be caused by a complex pathology, including abnormalities in the bones associated with hearing. Unfortunately, computed tomography imaging of the head was not performed in this patient, but it is conceivable that his hearing was improved owing to ossification of the auditory ossicles after asfotase alfa treatment.

Infants and young children show rapid and substantial improvements in skeletal mineralization, followed by improvements in respiratory, motor, and cognitive function after 1 year of treatment with asfotase alfa [9]. Our patient started asfotase alfa therapy at the age of 5 years and required a longer time to show treatment effects, such as bone mineralization, skeletal growth, and time until becoming ventilator free, compared with patients who started treatment when they were 3 years old [10]. In addition, it took 3 years to observe an improvement in hearing in our patient. In contrast, Okazaki et al. [6] reported a patient with perinatal HPP who started asfotase alfa therapy on day 1 after birth and showed improved hearing at 18 months. Our case demonstrated that bone mineralization, respiratory function, and hearing can be improved even when enzyme replacement therapy is started at a later time point. However, a delay in treatment causes a delay in the time until the effects of treatment are observed. Therefore, asfotase alfa administration should be started as early as possible after the diagnosis, to improve the patient's symptoms. Further studies are required to clarify the efficacy of asfotase alfa treatment for perinatal HPP.

## Funding

This research did not receive any specific grants from funding agencies in the public, commercial, or not-for-profit sectors.

## Declaration of Competing Interest

There is no conflict of interest.

## References

[1] Whyte M.P., Rockman-Greenberg C., Ozono K. (2016). Asfotase alfa treatment improves survival for perinatal and infantile hypophosphatasia. J. Clin. Endocrinol. Metab..

[2] Kishnani P.S., Rush E.T., Arundel P. (2017). Monitoring guidance for patients with hypophosphatasia treated with asfotase alfa. Mol. Genet. Metab..

[3] Colazo J.M., Hu1 J.R., Dahir K.M. (2019). Neurological symptoms in Hypophosphatasia. Osteoporos. Int..

[4] Takeshi T., Onigata K., Kobayashi H. (2014). Clinical and genetic aspects of hypophosphatasia in Japanese patients. Arch. Dis. Child..

[5] Al-Shawafi H.A., Komaru K., Oda K. (2017). Molecular defect of tissue-nonspecific alkaline phosphatase bearing a substitution at position 426 associated with hypophosphatasia. Mol. Cell. Biochem..

[6] Okazaki Y., Kitajima H., Mochizuki N. (2016). Lethal hypophosphatasia successfully treated with enzyme replacement from day 1 after birth. Eur. J. Pediatr..

[7] Cunningham C., Scheuer L., Black S. (2017). Developmental juvenile osteology, Chapter 5. The Skull.

[8] Bartel-Friedrich S., Wulke C. (2007). Classification and diagnosis of ear malformation. GMS. Curr. Top. Otorhinolaryngol. Head. Neck. Surg..

[9] Whyte M.P., Greenberg C.R., Salman N.J. (2012). Enzyme-replacement therapy in life-threatening hypophosphatasia. N. Engl. J. Med..

[10] Whyte M.P., Simmons J.H., Moseley S. (2019). Asfotase alfa for infants and young children with hypophosphatasia: 7 year outcomes of a single-arm, open-label, phase 2 extension trial. Lancet. Diabetes. Endocrinol..

